# Functional characterization of the diatom cyclin-dependent kinase A2 as a mitotic regulator reveals plant-like properties in a non-green lineage

**DOI:** 10.1186/s12870-015-0469-6

**Published:** 2015-03-14

**Authors:** Marie JJ Huysman, Atsuko Tanaka, Chris Bowler, Wim Vyverman, Lieven De Veylder

**Affiliations:** Department of Plant Systems Biology, Flanders Institute for Biotechnology (VIB), 9052 Ghent, Belgium; Department of Plant Systems Biology, VIB, and Bioinformatics, Ghent University, 9052 Ghent, Belgium; Protistology and Aquatic Ecology, Department of Biology, Ghent University, 9000 Ghent, Belgium; Environmental and Evolutionary Genomics Section, Institut de Biologie de l’Ecole Normale Supérieure, Centre National de la Recherche Scientifique, Unité Mixte de Recherche 8186, Institut National de la Santé et de la Recherche Médicale U1024, Ecole Normale Supérieure, 75230 Paris, Cedex 05 France; Current address: Muroran Marine Station, Field Science Center for Northern Biosphere, Hokkaido University, Muroran, Hokkaido 051-0013 Japan

**Keywords:** Cyclin-dependent kinase, CDKA2, Cell cycle, Cell division, Cytokinesis, Diatom, Mitosis, *Phaeodactylum tricornutum*

## Abstract

**Background:**

Cyclin-dependent kinases (CDKs) are crucial regulators of cell cycle progression in eukaryotes. The diatom *CDKA2* was originally assigned to the classical A-type CDKs, but its cell cycle phase-specific transcription at the G2-to-M phase transition is typical for plant-specific B-type CDKs.

**Results:**

Here, we report the functional characterization of CDKA2 from the diatom *Phaeodactylum tricornutum*. Through a yeast two-hybrid library screen, CDKA2 was found to interact with the G2/M-specific CDK scaffolding factor CKS1. Localization of CDKA2 was found to be nuclear in interphase cells, while in cells undergoing cytokinesis, the signal extended to the cell division plane. In addition, overexpression of *CDKA2* induced an overall reduction in the cell growth rate. Expression analysis of cell cycle marker genes in the overexpression lines indicates that this growth reduction is primarily due to a prolongation of the mitotic phase.

**Conclusions:**

Our study indicates a role for CDKA2 during cell division in diatoms. The functional characterization of a CDK with clear CDKB properties in a non-green organism questions whether the current definition of B-type CDKs being plant-specific might need revision.

**Electronic supplementary material:**

The online version of this article (doi:10.1186/s12870-015-0469-6) contains supplementary material, which is available to authorized users.

## Background

In eukaryotes, control of cell cycle progression is driven by an evolutionarily conserved family of serine/threonine kinases, the cyclin-dependent kinases (CDKs). CDKs form functional heterodimers with regulatory cyclin subunits [1,2]. Together, CDKs and their cyclin partners control cell cycle progression at the G1 (Gap 1)-to-S (Synthesis) and the G2 (Gap 2)-to-M (Mitosis) phase transitions through the phosphorylation of target proteins involved in DNA replication and mitosis, respectively [3]. The activity of CDK/cyclin complexes is regulated at multiple levels, including the interaction with inhibitors or scaffolding proteins, and phosphoregulation of the CDK subunit.

In contrast to fission yeast (*Schizosaccharomyces pombe*) and budding yeast (*Saccharomyces cerevisiae*), in which only a single CDK (Cdc2/Cdc28) controls the cell cycle [4,5], animals and plants possess multiple CDKs [1,6]. The most conserved cell cycle regulators possess a typical PSTAIRE cyclin-binding motif (Cdc2/Cdc28 in yeast, Cdk1/Cdk2 in animals and A-type CDKs in plants). In addition, a class of CDKs specific to plants has been shown to control the cell cycle, being called the B-type CDKs [7-10]. B-type CDKs in higher plants possess a variant of the PSTAIRE motif, either PPTALRE (CDKB1) or PPTTLRE (CDKB2) [6] and, unlike the A-type CDKs that are required for both the G1-to-S and G2-to-M phase transition, they only play a role at the G2/M boundary [11].

With the recent advances in sequencing techniques, more genomes have become available, including several from different algal groups [12]. This wealth of new data makes it possible to study B-type CDK evolution by comparative genomics. CDKB-like sequences have been identified in different algal species, including the green algae *Ostreococcus tauri* [13], *Chlamydomonas reinhardtii* [14], *Micromonas sp.* and *Micromonas pusilla*, the red alga *Cyanidioschyzon merolae* [15], and also the brown alga *Ectocarpus siliculosus* [16]. Remarkably, the CDKB-like sequences of *O. tauri* and *C. reinhardtii* have been reported to represent functional homologs of A-type CDKs, mainly by their ability to complement *cdc28* temperature-sensitive yeast mutants [15,17], indicating that B-type CDKs might originate from a duplication and subsequent specification of the A-type CDKs [18]. However, to date, their functionality to complement higher plant B-type CDKs has not been investigated.

*Phaeodactylum tricornutum* is a unicellular marine diatom belonging to the heterokont (or stramenopile) lineage [19]. This diatom multiplies by binary division and, unlike most other diatoms, it lacks a sexual phase during its life cycle, rendering this diatom a perfect model species to study vegetative reproduction [20-23]. Furthermore, due to the presence of a light-dependent phase during its cell cycle [21,24], the cell division process in *P. tricornutum* can easily be synchronized by implementation of alternating light/dark cycles [21]. Phylogenetic analysis of all CDKs identified in *P. tricornutum* revealed the presence of two A-type CDKs, of which CDKA1 shows the classical PSTAIRE motif, while CDKA2 shows the divergent PSTALRE cyclin-binding motif [21]. The latter deviates only by one amino acid from the CDKA (PSTAIRE) and CDKB (P[P/S]T[A/T]LRE) hallmarks. Such a PSTALRE motif is also present in the *Dictyostelium discoideum* CDC2 homolog (DdCDK1) [25], the *C. merolae* CDKA protein [15], the *O. tauri* CDKB protein [13] and the *E. siliculosus* CDKA2 protein [16]. Moreover, unlike typical CDKAs, transcription of the *P. tricornutum CDKA2* gene is cell cycle-regulated and shows a peak of transcription at the G2-to-M phase transition [21]. However, based on its current phylogenetic position and its transcription pattern, it is impossible to define whether *CDKA2* represents a functional ortholog of A- or B-type CDKs.

The main objective of this study was to functionally elucidate the role of CDKA2 during the cell cycle in *P. tricornutum*. Overexpression of *CDKA2* resulted in a delayed growth phenotype, mainly at the G2/M phase of the cell cycle. Moreover, microscopic analysis of cells expressing a fluorescently-tagged version of CDKA2 showed a relocalization of CDKA2 from the nucleus to the cell division plane just before cytokinesis. Together, these data suggest a role for CDKA2 during mitosis in diatoms.

## Results

### Phylogenetic analysis of CDKA2

**Figure 1:**
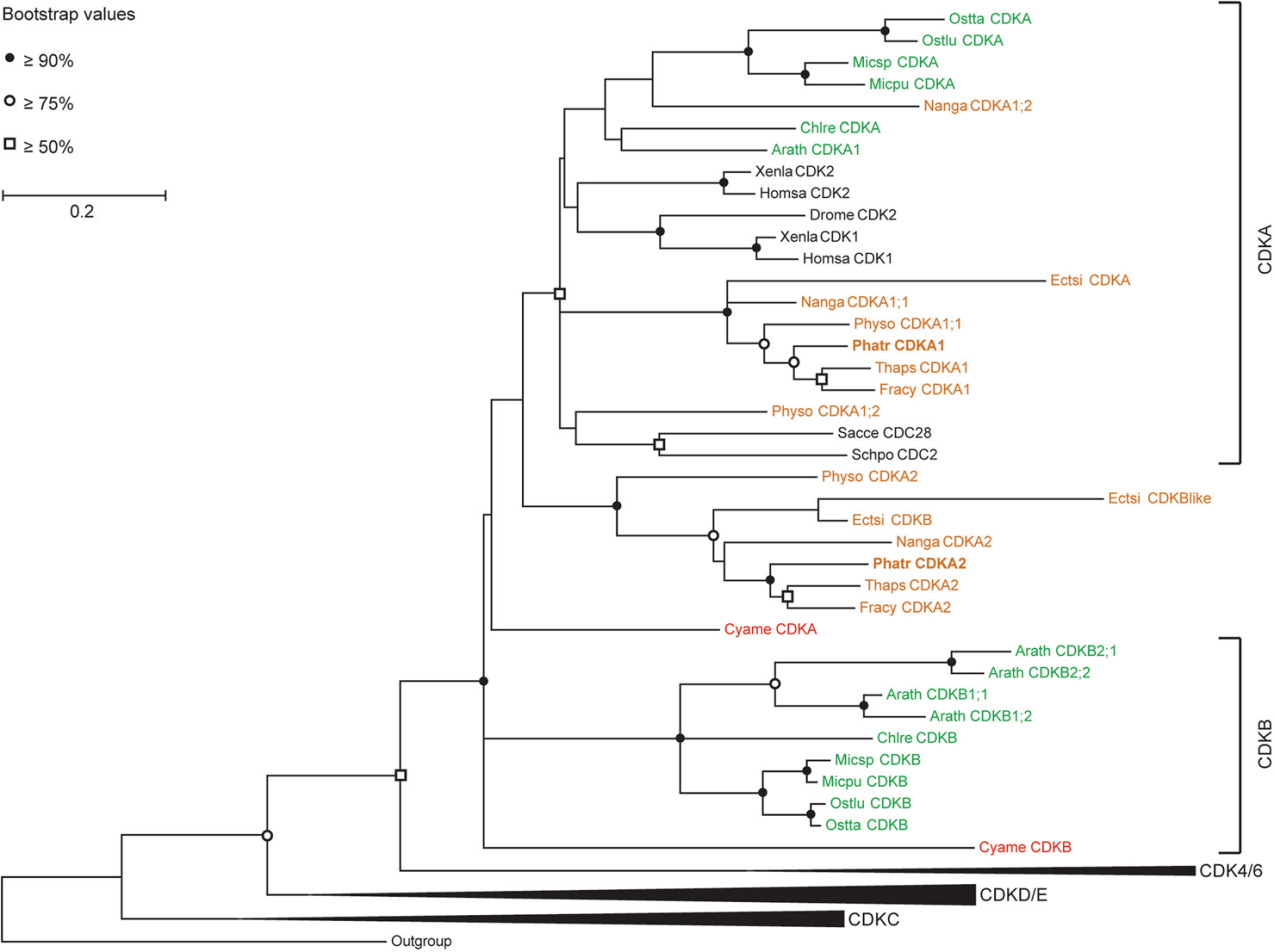
**Phylogenetic analysis of the cyclin-dependent kinases of** ***P. tricornutum*** **.** Maximum-likelihood tree (MEGA5.1, 1000 replicates) of the CDK family. The *P. tricornutum* sequences are shown in bold. Stramenopile sequences are shown in brown, higher plant and green algal sequences are indicated in green, red algal sequences in red and animal or yeast sequences in black. Abbreviations: Arath, *Arabidopsis thaliana*; Chlre, *Chlamydomonas reinhardtii*; Cyame, *Cyanidioschyzon merolae*; Drome, *Drosophila melanogaster*; Ectsi, *Ectocarpus siliculosus*; Fracy, *Fragilariopsis cylindrus*; Homsa, *Homo sapiens*; Micpu, *Micromonas pusilla*; Micsp, *Micromonas sp.*; Nanga, *Nannochloropsis gaditana*; Ostlu, *Ostreococcus lucimarinus*; Ostta, *Ostreococcus tauri*; Phatr, *Phaeodactylum tricornutum*; Physo, *Phytophthora sojae*; Sacce, *Saccharomyces cerevisiae*; Schpo, *Schizosaccharomyces pombe*; Thaps, *Thalassiosira pseudonana* and Xenla, *Xenopus laevis*. The outgroup is represented by human CDK10.

Considering the ambiguous classification of CDKA2, its phylogenetic position among the CDK family members was reanalyzed (Figure 1). The recent release of genome data from several stramenopile algae, including the diatom *Fragilariopsis cylindrus*, the brown alga *E. siliculosus* [26] and the eustigmatophyte alga *Nannochloropsis gaditana* [27], allowed us to include CDK sequences of these species in the multiple sequence alignment. In the updated phylogenetic tree, the diatom CDKA2 sequences group together with CDKA2 and CDKB-like sequences from the other stramenopile groups (*E. siliculosus*, *N. gaditana* and *Phytophthora sojae*) as a well-supported clade that may represent a stramenopile-specific cluster. Although this cluster is clearly distinct from the plant B-type CDK cluster, it does not belong to the archetypical A-type CDK cluster either. Based on this updated phylogenetic analysis, it therefore remains impossible to determine whether CDKA2 is more related to the A-type or B-type CDKs (Figure 1).

### Cell cycle phase-dependent expression of *CDKA2*

**Figure 2:**
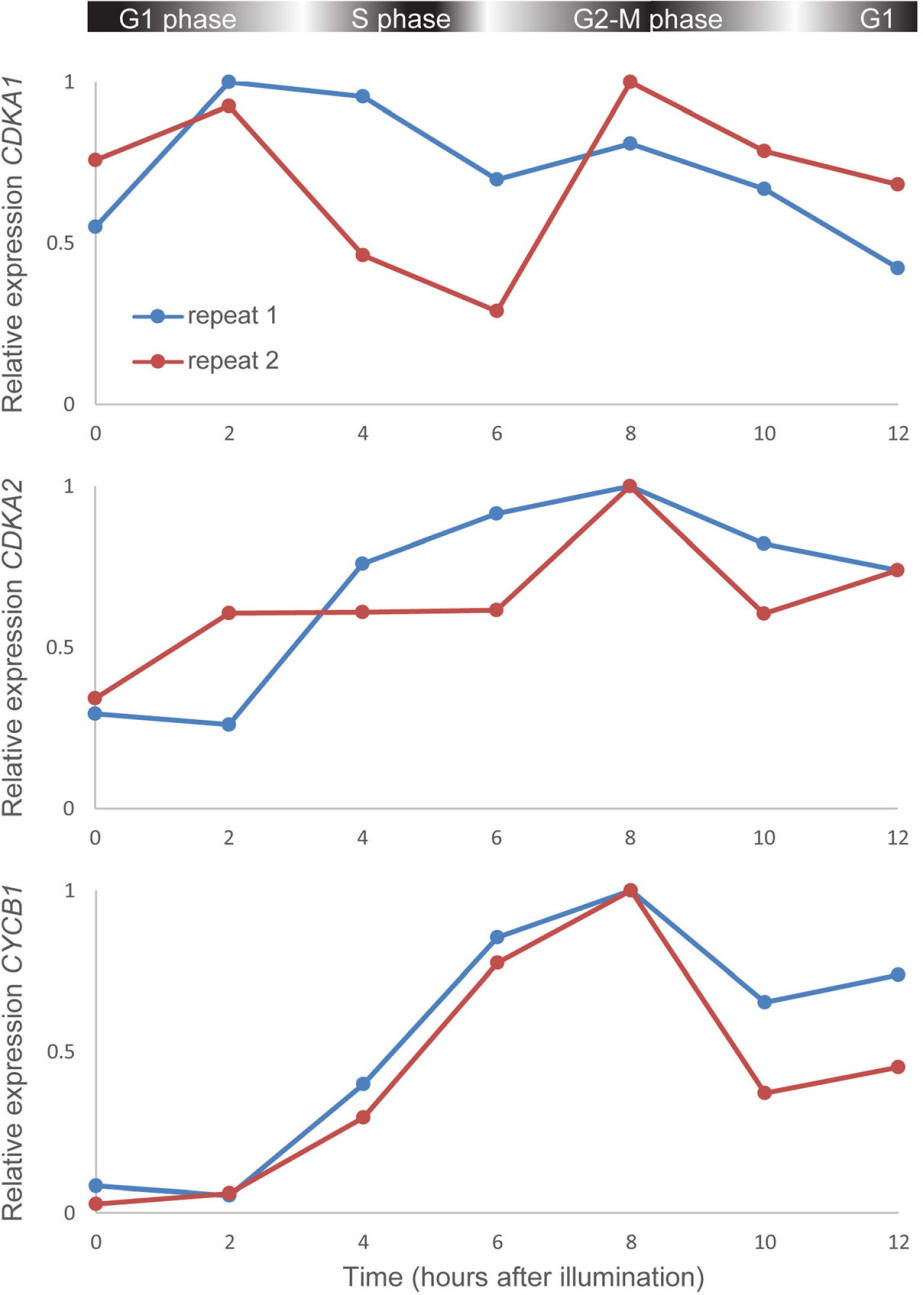
**Transcript expression profiles of** ***CDKA1*** **and** ***CDKA2*** **during the cell cycle in wild-type** ***P. tricornutum*** **cells.** Transcript levels were measured by nCounter analysis, normalized using four reference genes (*EF1a*, *histone H4*, *RPS*, and *UBI-4*) and rescaled to the maximum expression value (=1). Approximate cell cycle phase timing for this sample series was reported previously [22] and is indicated at the top.

Previous work suggested that *CDKA2* transcription is cell cycle-regulated and shows a peak at the G2-to-M phase transition [21]. However, the experimental setup used in that study did not allow assessing expression levels beyond the metaphase point, since a microtubule depolymerizing agent, nocodazole, was added to the cells to increase the proportion of cells at mitosis. Here, we further analyzed the temporal expression of *CDKA1* and *CDKA2* during the cell cycle in synchronized *P. tricornutum* cells in the absence of nocodazole (Figure 2). In contrast to *CDKA1*, which displays no reproducible temporal expression profile during the cell cycle, *CDKA2* levels start accumulating during the G1/S phase and reach a maximum peak at the G2/M phase, coinciding with the peak expression of the mitotic marker *cyclin B1* (*CYCB1*).

### CDKA2 interacts specifically with CKS1

To identify putative interactors of the *P. tricornutum* CDKA proteins, yeast two-hybrid (Y2H) cDNA library screens were conducted using the full-length CDKA1 and CDKA2 sequences fused to the GAL4 DNA-binding domain as bait. Using CDKA2 as bait, four possible interacting proteins were detected (Additional file 1: Table S1). However, only one of these interactors (Cdc Kinase Subunit 1, CKS1) could be confirmed (Additional file 2: Figure S1). Previously, we have shown that *CKS1* is predominantly transcribed during the G2/M phases, coinciding with the expression pattern of *CDKA2* [21]. Using CDKA1 as bait, 84 clones were isolated, representing 12 different putative interacting proteins (Additional file 1: Table S1). Of these, three belong to the cyclin family (cyclin P1 (CYCP1), cyclin P6 (CYCP6) and diatom-specific cyclin 7 (dsCYC7)) [21].

**Figure 3:**
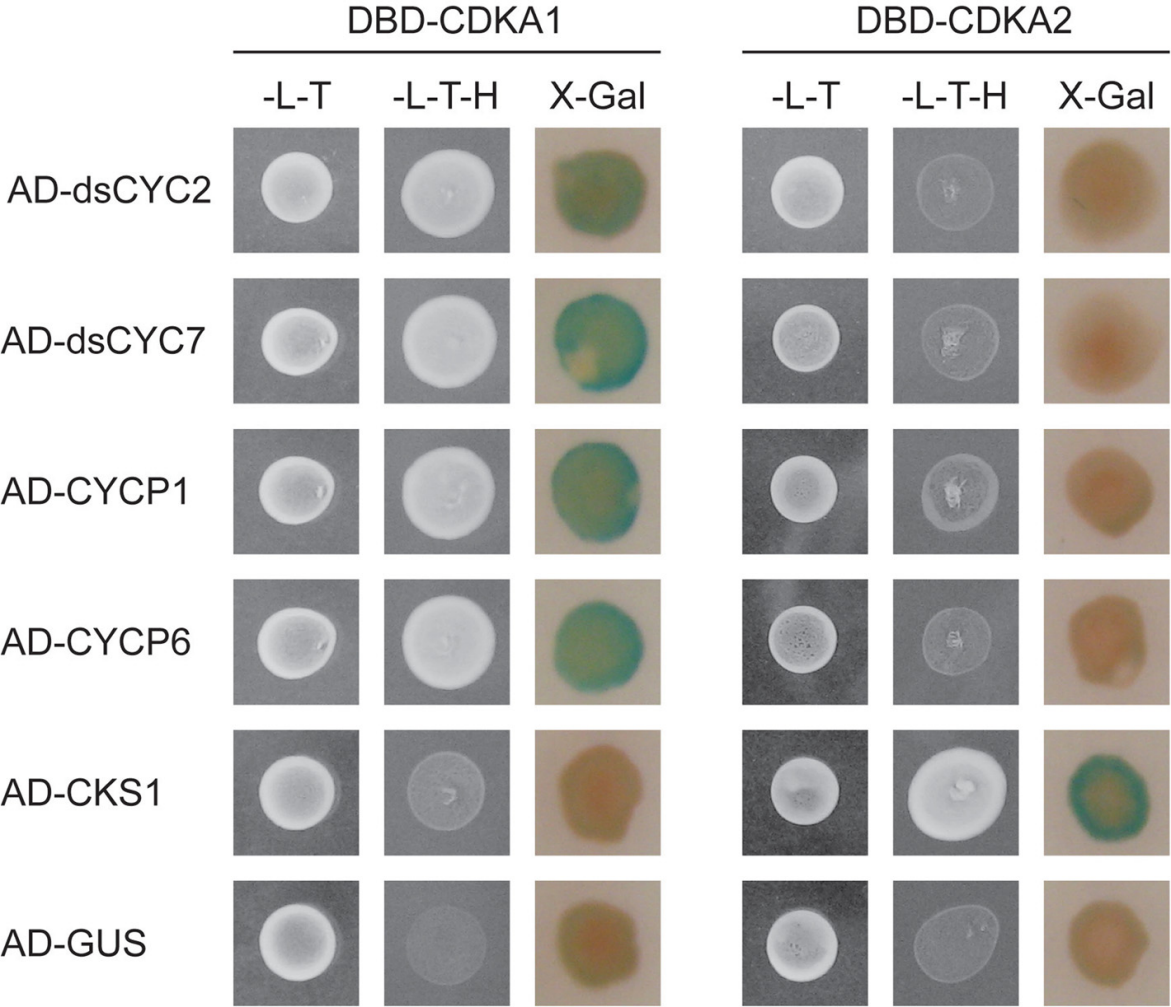
**Interactions with CDKA1 and CDKA2 proteins using pairwise Y2H co-transformation assays.** Yeast PJ694-alpha cells were co-transformed with bait (DBD) and prey (AD) plasmid as indicated. Co-transformation was analyzed on medium lacking leucine and tryptophan (-L-T). Co-transformants were tested for their ability to activate the histidine marker gene by assessing yeast growth on medium lacking leucine, tryptophan and histidine (-L-T-H) and for their ability to activate the *LacZ* reporter gene (X-Gal). As a negative control, the *GUS* gene was used. For each combination, three independent colonies were screened, of which one is shown.

To test the binding specificity of the two baits, pairwise Y2H assays were performed by co-transforming them in yeast with the prey proteins CKS1, CYCP1, CYCP6, dsCYC7 and dsCYC2, which was previously described as an interactor of CDKA1 [28]. Interestingly, CDKA1 was not able to bind CKS1 in the Y2H assay, whereas CDKA2 did not interact with any of the cyclins tested (Figure 3), indicating that the interactions picked up by Y2H library screens are specific for each CDK.

### Overexpression of *CDKA2* reduces the cell growth rate by interfering with G2/M phase progression

**Figure 4:**
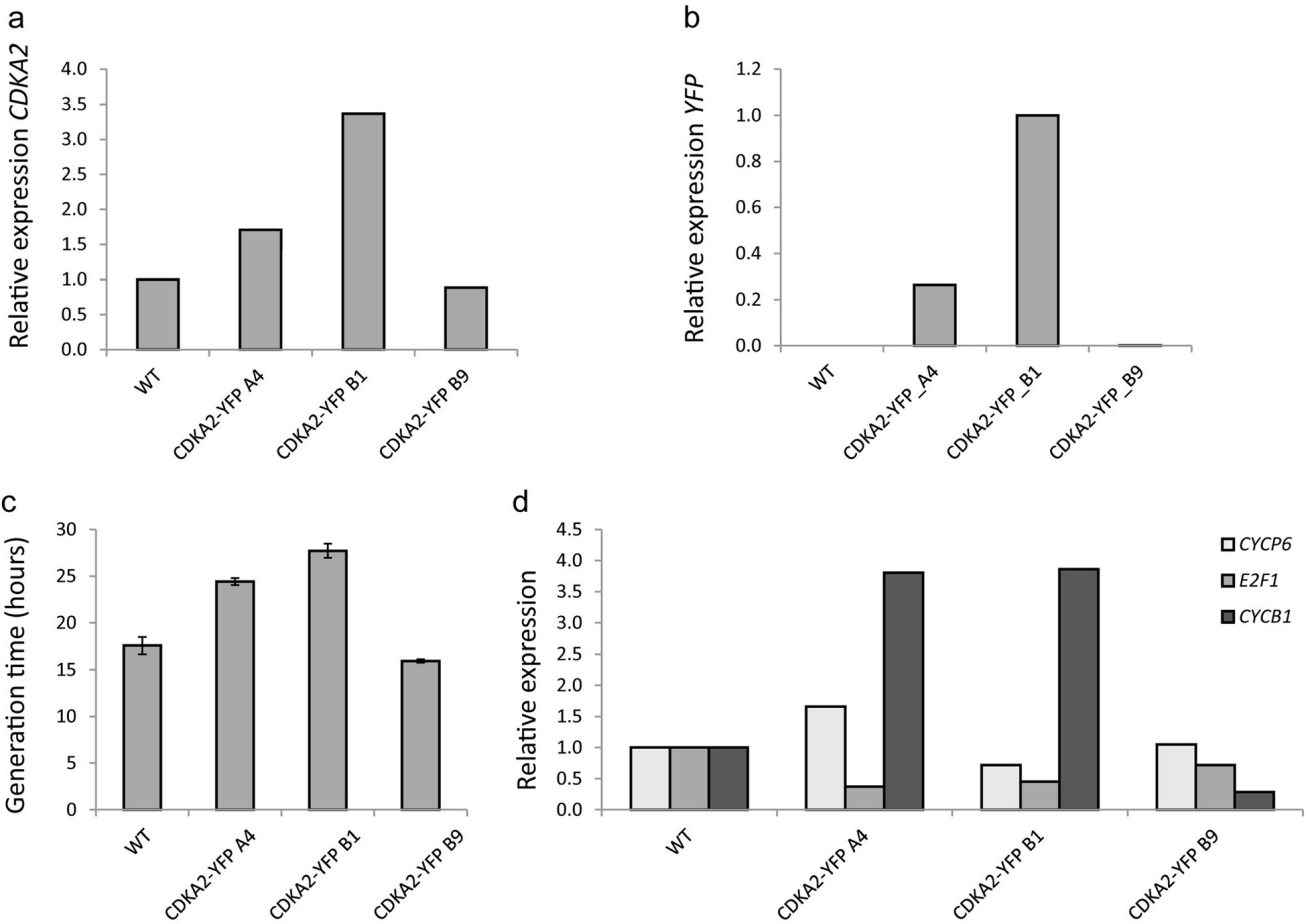
**Effect of** ***CDKA2-YFP*** **overexpression on cell cycle progression in** ***P. tricornutum*** **. (a)** Real-time qPCR analysis of *CDKA2* transcript levels in WT and transgenic lines. **(b)** Real-time qPCR analysis of *YFP* transcript levels in WT and transgenic lines. **(c)** Generation times of overexpression (A4 and B1) and control lines (WT and B9) grown under constant light. Error bars represent standard errors of the mean of three biological replicates. **(d)** Real-time qPCR analysis of different cell cycle marker genes in overexpression (A4 and B1) and control lines (WT and B9).

To study the function of CDKA2 during the cell cycle of *P. tricornutum*, the effect of *CDKA2* overexpression on cell cycle progression was determined. Transgenic lines were generated that overexpress *CDKA2* fused at its C-terminus to *yellow fluorescent protein* (*YFP*) (*CDKA2-YFP*) under control of the *fucoxanthin chlorophyll binding protein B* (*fcpB*) promoter. Overexpression of *CDKA2-YFP* was evaluated by transcript analysis of *CDKA2* (Figure 4a) and *YFP* (Figure 4b) using real-time quantitative PCR (RT qPCR) in wild-type (WT) and transgenic cells. Two lines expressed an approximately two- to three-fold higher level of *CDKA2* transcripts compared to WT cells (CDKA2-YFP A4 and CDKA2-YFP B1, respectively) (Figure 4a) due to the overexpression of the *CDKA2-YFP* fusion (Figure 4b), while another line (CDKA2-YFP B9) showed no increase of *CDKA2* (Figure 4a). In contrast to the CDKA2-YFP A4 and B1 lines, no expression of *YFP* could be detected for the CDKA2-YFP B9 line (Figure 4b). This line was therefore used as an internal transformation control in downstream analyses.

To determine if *CDKA2* overexpression alters cell cycle dynamics, a growth analysis was performed. Optical density determination, which can be used as an estimate of cell number, was performed in the morning during nine consecutive days, in lines grown under constant light conditions. Cells expressing increased levels of *CDKA2-YFP* (CDKA2-YFP A4 and CDKA2-YFP B1) showed longer generation times compared to the control cells (WT and CDKA2-YFP B9) (Figure 4c). To determine which cell cycle phase was affected, we measured the transcript levels of *CYCP6*, *E2F1* and *CYCB1* in exponentially growing asynchronous *CDKA2-*overexpressing and control cells. These genes represent diatom cell cycle marker genes specific for the G1, S and G2/M phases, respectively [21,28]. In both *CDKA2* overexpression lines, we detected higher transcript levels of the mitotic marker *CYCB1*, and slightly lower *E2F1* levels compared to those of the control cells (Figure 4d), indicating that *CDKA2*-overexpressing cells spend more time at the G2/M phase.

### CDKA2 relocalizes from the nucleus to the division plane before cytokinesis

**Figure 5:**
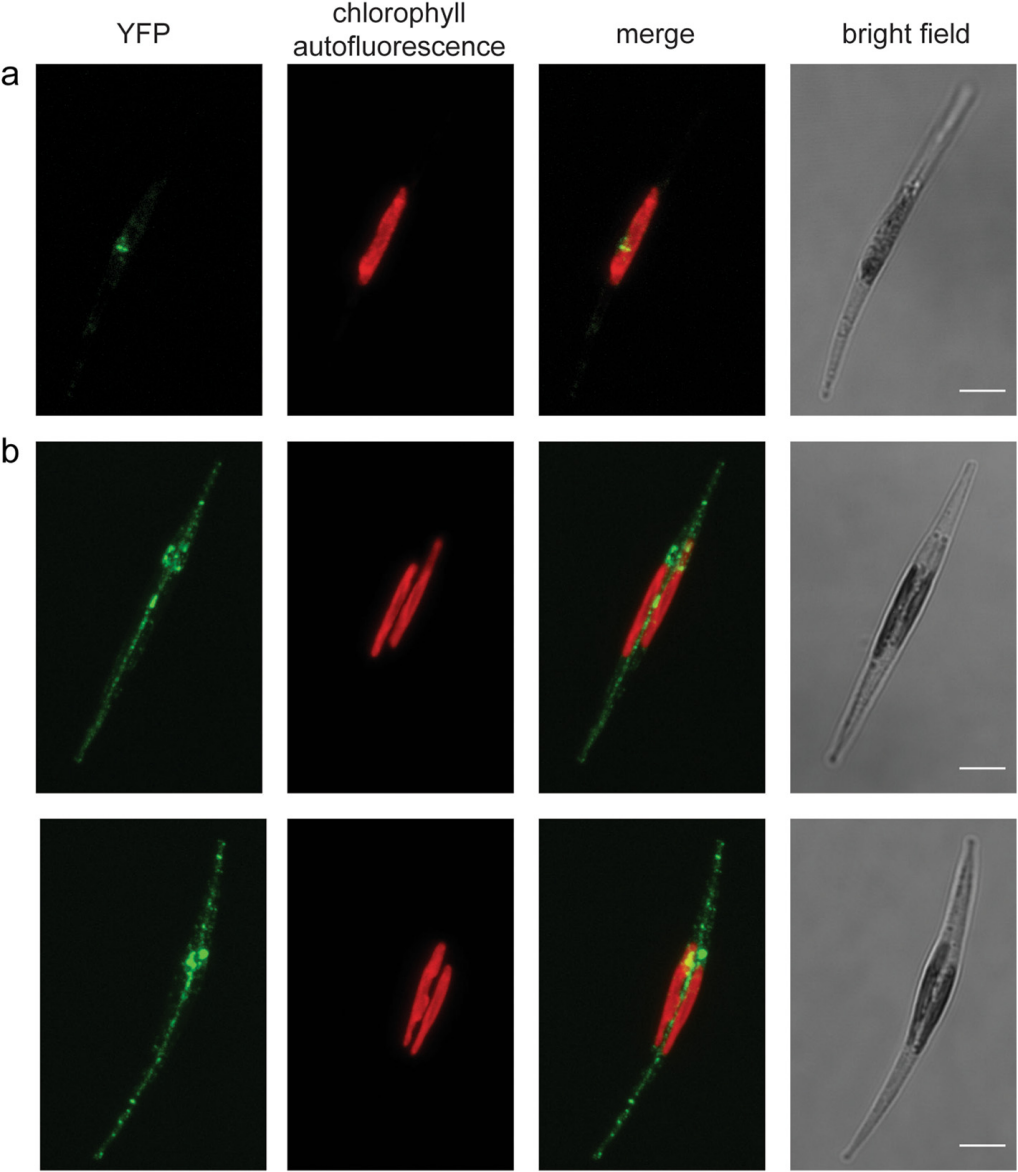
**Localization of CDKA2-YFP in** ***P. tricornutum*** **.** Confocal images of *CDKA2-YFP* overexpressing cells during different stages of the cell cycle. Maximum intensity Z-projections of CLSM analyses acquired with a Leica SP5 device are shown. For the bright field panels, a representative single plane is shown. **(a)** Interphase cell with undivided chloroplast. **(b)** Cells during cytokinesis with divided chloroplast and nucleus. At the time of cytokinesis, the signal was no longer prominent in the nucleus, but it was also targeted to the plane of division. The YFP signal is indicated in green and chlorophyll autofluorescence from the chloroplast in red. Scale bars represent 5 μm.

To explore the subcellular localization of CDKA2 during the cell cycle, we monitored YFP fluorescence in the *CDKA2-YFP* overexpression cells. To avoid aberrant localization patterns due to the overexpression of *CDKA2*, we selected the CDKA2-YFP A4 line for this analysis, which shows only about two-fold higher expression levels of *CDKA2* (Figure 4a). For microscopic observations, cells were synchronized to enrich for cells at the different phases of the cell cycle, including interphase and mitosis. Confocal laser-scanning microscopy (CLSM) revealed a predominant nuclear and weak cytosolic YFP fluorescence in interphase cells that contain undivided or divided translocating chloroplasts (Figure 5a). This predominant nuclear localization of the YFP signal was confirmed by co-localization of Hoechst33342 DNA staining (Additional file 3: Figure S2). Remarkably, in cells with fully translocated daughter chloroplasts, the YFP fluorescence extended to the cell division plane between the two daughter chloroplasts and resembled dot-like signals (Figure 5b). Three-dimensional reconstruction of the Z-stack of confocal images clearly demonstrated the localization of CDKA2-YFP fluorescence to a plate-like structure (Additional files 4: Movie S1 and 5: Movie S2). Following division, the YFP signal is localized in the nucleus of both daughter cells.

## Discussion

In this study, we provide experimental evidence elucidating the role of CDKA2 in the diatom *P. tricornutum*, a CDK previously assigned to the A-type family, but displaying a PSTALRE motif, a motif in-between those of the conserved A-type and plant-specific B-type CDKs [6,21]. In contrast to classical A-type CDKs, transcription of diatom *CDKA2* fluctuates during the cell cycle, increasing from the G1/S phase onward and showing a peak in its expression at mitosis, thus resembling the expression pattern of B-type CDKs [29,30] and suggesting a role for CDKA2 in the control of mitosis. Re-examination of the phylogenetic position of *CDKA2*, by including closely related organisms, assigned it to a stramenopile cluster of CDKA/B-like sequences. The phylogenetic tree indicates a rapid radiation of CDK types near the base of the CDKA and CDKB lineage, possibly representing a *CDK* gene diversification phase during the early stages of eukaryotic evolution. The hypothesis that CDKA2 may function as a mitotic regulator is further supported by the localization of CDKA2-YFP at the cell division plane in pre-cytokinetic cells. This localization pattern suggests that CDKA2 may play a role in the positioning of the cleavage furrow or the formation of cytokinetic structures, or in the recruitment of one or various components to the cell division plane. In mammals, the activity of PRC1, a microtubule (MT) binding and bundling protein responsible for MT stabilization during cytokinesis, is regulated through phosphorylation by CDKs [31]. Also in plant cells, MT-dependent association of CDKs with mitotic structures and the functional involvement of CDKs in the organization of specific MT arrays have been reported [32-36].

CDKA2 was found to interact with CKS1, whose gene was previously reported to be transcribed mainly at the G2/M phase in synchronized cells [21]. The interaction with CKS1 was not surprising, since CKS1 is a member of a conserved family of small proteins that are believed to act as docking factors that mediate the interaction of CDKs with regulatory proteins and putative substrates [37]. CKS1 might therefore represent a good bait to identify putative substrates of CDKA2 in further studies, which might help us to determine the precise function and action of CDKA2 during diatom mitosis. Interestingly, CKS1 was found not to bind CDKA1, resembling the situation in animal cells, in which CKS proteins have been reported to bind to Cdk2 complexes, but not to the G1/S-specific Cdk4/6 complexes [37-39].

In plants, overexpression of wild-type *CDK* genes generally does not trigger a phenotype [7,8,40]. However, recently, the overexpression of *CDKA1* in tomato fruit was reported to cause an increase in cell division [41], while the overexpression of *CDKB1* or *CDKB2* reduces the cell division rate in tomato fruit pericarp [42]. In addition, overexpression of *CDKB2;1* and *CDKB2;2* in *Arabidopsis* reduces cell cycle progression in the meristem due to regulatory defects at the G2-to-M transition [43]. Therefore, overexpression of B2-type *CDK*s appears to induce a consistent cell cycle phenotype, suggesting a universal role for these CDKs during cell cycle regulation. Here, we observed major growth defects in *P. tricornutum* cells overexpressing *CDKA2*. Most prominently, cell generation time almost doubled, despite only a slight increase in *CDKA2* transcript levels. Analysis of the expression of cell cycle marker genes demonstrated that this delay most probably results from a lengthening of the G2/M phases, as *CYCB1* levels were clearly elevated in *CDKA2*-overexpressing cells. Several hypotheses could explain the *CDKA2* overexpression phenotype. First, the overexpression construct could compete with endogenous *CDK* genes for rate-limiting interacting proteins (e.g., mitotic cyclins or CKS1). On the other hand, the mitotic arrest observed in the *CDKA2*-overexpressing cells might result from aberrant levels and timing of CDKA2 activity. In eukaryotes, exit from mitosis strictly depends on the downregulation of CDK activity, which is initiated by the destruction of mitotic cyclins by the anaphase promoting complex/cyclosome (APC/C) [44,45]. In a previous study, we have identified all essential components of this ubiquitin ligase complex in *P. tricornutum* and reported the cell cycle phase-specific transcription of its activators CDC20 and CDH1 [22], suggesting that a similar control mechanism for mitotic exit by a decline in CDK activity might exist in diatoms.

In yeast and metazoans, CDC25 phosphatases are known to activate CDKs by opposing the activity of the WEE1/MYT1/MIK1 family of inhibitory kinases [46]. Phosphorylation of conserved Thr (T14) and/or Tyr (Y15) residues results in CDK inactivation upon activation of the cell cycle checkpoints caused by triggers that should stop the cell cycle, such as DNA damage or mitotic defects. Dephosphorylation of these residues by CDC25 renders the CDK/cyclin complex active and hence stimulates cell cycle progression [47]. Although Tyr phosphorylation in plants is important to arrest the cell cycle under stress conditions, it does not seem to be crucial for G2-to-M progression [40,48-50]. Interestingly, all organisms in which B-type CDKs have been identified appear to lack a functional CDC25 phosphatase [13,51]. The only exception is *O. tauri*, which contains both a CDC25 phosphatase and a B-type-like CDK. However, functional analysis of the *O. tauri* CDKB indicated some CDKA features, including the ability to complement a yeast *cdc28* mutant, suggesting that this protein is rather atypical and has not yet achieved all the functional properties of higher plant B-type CDKs [17]. Because of an intriguing number of parallels in transcriptional, biochemical and functional properties of mammalian CDC25 and plant B-type CDKs, it was suggested that the CDC25-mediated regulatory mechanisms might have been replaced in plants by a mechanism governed by the plant-specific B-type CDKs [18]. Despite the presence of almost all regulatory components of the eukaryotic cell cycle in *P. tricornutum*, diatoms also lack a clear CDC25 phosphatase homolog [21], which may explain the need for a CDKB-like CDK, such as CDKA2, controlling the G2-to-M progression.

## Conclusions

In this study we addressed the ambiguous nature of CDKA2 in *P. tricornutum*. CDKA2 was originally assigned to the A-type CDKs, but displays some typical characteristics of the plant-specific B-type CDKs, including cell cycle phase-dependent transcription at the G2-to-M transition. Subcellular localization of CDKA2 at the cell division plane during cytokinesis and its interaction with the G2/M expressed cell cycle regulator CKS1, point to a function for CDKA2 during mitosis. In addition, *CDKA2* overexpression resulted in the prolongation of the mitotic phase and an increase in cell cycle duration, demonstrating its role as a mitotic regulator. This is the first functional characterization of a CDK with clear CDKB properties in a non-green lineage, indicating that, while B-type CDKs form a clear plant-specific clade at the phylogenetic level, functional orthologs can be identified in other eukaryotic groups.

## Methods

### Phylogenetic analysis

Sequence data was retrieved from the NCBI RefSeq database (*A. thaliana*, *C. merolae*, *C. reinhardtii*, *D. melanogaster*, *H. sapiens*, *N. gaditana*, *P. sojae*, *S. cerevisiae*, *S. pombe* and *X. laevis*), through the JGI portal (*P. tricornutum* and *T. pseuodonana*) or the pico-PLAZA database (*E. siliculosus*, *F. cylindrus*, *M. pusilla*, *M. sp.*, *O. tauri* and *O. lucimarinus*) [12,52]. Multiple alignments based on CDK amino acid sequences were generated with MUSCLE [53] and then manually improved, yielding 226 amino acid positions (Additional file 6: Figure S3). To define subclasses within the gene families, phylogenetic trees were built that included reference CDK sequences from animals, yeast, higher plants, stramenopiles and several algal species. MEGA5.1 was used to construct the maximum-likelihood tree using the rtREV + G + I model. To test the significance of the nodes, bootstrap analysis was applied using 1,000 replicates.

### Diatom culture conditions

*Phaeodactylum tricornutum* (Pt1 8.6; accession numbers CCAP 1055/1 and CCMP2561) was grown in f/2 medium without silica (f/2-Si) [54] made with autoclaved filtered sea water. For routine cultivation, cells were grown at 18-20°C in a 12-h light/12-h dark regime and 70–100 μmol photons m^−2^ s^−1^. Liquid cultures were shaken at 100 rpm. For biolistic transformation, *P. tricornutum* cells were grown on solid f/2-Si medium containing 1% Select agar (Sigma).

### nCounter analysis

The sample series that was used to address the cell cycle phase-dependent transcription of *CDKA1* and *CDKA2* in wild-type *P. tricornutum* cells was described elsewhere [22,28]. Synchronization, RNA extraction and Nanostring nCounter analysis (Nanostring Technologies) were performed as described before [22,28]. Briefly, transcript levels were measured in multiplexed reactions using the nCounter analysis system by the VIB MicroArrays Facility (www.microarrays.be) as described before [55] and normalized using four reference genes (*EF1a*, *histone H4*, *RPS*, and *UBI-4*). Additional file 7: Table S2 gives an overview of the nCounter probe pairs used in this study. The analysis was done using two biological replicates.

### Yeast two-hybrid analysis

Yeast two-hybrid bait plasmids were generated through recombinational GATEWAY cloning (Invitrogen). To generate the bait ENTRY clones, full-length open reading frames of the *P. tricornutum CDKA1* and *CDKA2* were isolated and cloned into the pENTR-D-TOPO vector as described elsewhere [28]. The obtained ENTRY clones were recombined in the pDEST32 (bait) vector (Invitrogen) by attL × attR recombination, resulting in translational fusions between the proteins and the GAL4 DNA-binding domains. Plasmids encoding the bait constructs were transformed in the yeast strain PJ694-alpha (MATa; trp1-901, leu2-3,112, ura3-52, his3-200, gal4D, gal80D, LYS2::GAL1-HIS3, GAL2-ADE2, met2GAL7-lacZ) by the LiAc method [56] to generate bait strains. Y2H library screens were performed using a custom-made Y2H cDNA library (Invitrogen) described before [28]. To this end, the respective bait strain was transformed with 50 μg of prey plasmids derived from the Y2H cDNA library according to the protocol described in the Yeast Protocol Handbook (Clontech). For each Y2H library screen at least 10^6^ transformants were screened. Putative positive Y2H interactions were selected on synthetic dextrose (SD) plates lacking Leu, Trp and His. Growing colonies were streaked on SD medium lacking Leu, Trp and His, and plasmid was purified from yeast patches using the Zymoprep I Yeast Plasmid Minipreparation Kit (Zymo Research) according to the manufacturers’ instructions. Yeast plasmid was used as a template in a PCR reaction with primers flanking the gateway cloning site of pDEST22 (pDEST22_Fw: TATAACGCGTTTGGAATCACT and pDEST22_Rv: AGCCGACAACCTTGATTGGAGAC), and the obtained PCR product was sequenced and blasted against the *P. tricornutum* genome database (http://genome.jgi-psf.org/Phatr2/Phatr2.home.html) to identify the putative interactor protein. To confirm the protein-protein interactions of interest, the respective prey and bait plasmids were co-transformed in the yeast strain PJ694-alpha, and the interactions were retested on medium lacking Leu, Trp and His and by X-Gal testing.

### Generation of the *CDKA2* overexpression construct

The full-length sequence of *CDKA2* was isolated and amplified by PCR using the CDKA2_Fw (CACCATGGAACGTTACCATAAGATAGAAAAG) and CDKA2-C_Rv (GATGTTTTCCTTATCCAAGTCATCA) primers designed to allow C-terminal fusion. The purified fragment was cloned into the pENTR-D-TOPO vector (Invitrogen) using the directional TOPO cloning strategy and the obtained ENTRY clone was subsequently recombined into pDEST-C-EYFP, a diatom adapted destination vector for C-terminal fusion with the YFP fluorescent marker under the control of the *fcpB* promoter [57], using the Gateway attL × attR recombination reaction (Invitrogen).

### Biolistic transformation

The CDKA2-YFP expression construct was introduced into *P. tricornutum* by microparticle bombardment as previously described [58]. As a selection marker, the pAF6 plasmid was co-transformed with the overexpression construct to confer resistance to phleomycin [57,58]. Transformants were initially selected based on their ability to grow on medium containing phleomycin (100 μg/ml final concentration). Individual resistant colonies were both restreaked on f/2-Si agar plates and grown in liquid f/2-Si medium without antibiotics for further analysis.

### Real-time quantitative PCR

Cells were grown in continuous light conditions to desynchronize the cells. For RNA extraction, 5 x 10^7^ exponentially growing cells were collected by centrifugation (15 minutes at 3,000 rpm, 4°C), fast frozen in liquid nitrogen and stored at −70°C. Cell lysis and RNA extraction was performed using TriReagent (Molecular Research Center, Inc., Cincinnati, OH, USA) according to the manufacturer’s instructions. Contaminating genomic DNA was removed by DNaseI treatment (Promega). To assess RNA concentration and purity, spectrophotometry was used (NaNodrop ND-1000, Wilmington, DE). Total RNA was reverse transcribed using iScript reverse transcriptase (Roche). Finally, 10 ng of cDNA was used as template in each qPCR reaction.

Samples in triplicate were amplified on the Lightcycler 480 platform with the Lightcycler 480 SYBR Green I Master mix (Roche Applied Science), in the presence of 0.5 μM gene-specific primers (YFP_Fw: TGCTTCGCCCGCTACCC and YFP_Rv: ATGTTGCCGTCCTCCTTGAAG; E2F1_Fw: CCCTAAGCGGCGGATTTACG; E2F1_Rv: AAGCGACGAGCCAAGAAGAAGC; other primers see [21]). The cycling conditions were 10 min polymerase activation at 95°C and 45 cycles at 95°C for 10 s, 58°C for 15 s and 72°C for 15 s. Amplicon dissociation curves were recorded after cycle 45 by heating from 65°C to 95°C. Data were analyzed using the qbase^+^ software package (Biogazelle) using the stably expressed *EF1a* and *TubA* as normalization genes [57].

### Growth analysis

To monitor growth, cells were grown at constant illumination in a 24-well plate (Falcon), in a total volume of 1 ml, over a time period of nine days. Absorbances of the cultures were measured at 405 nm using the VICTOR^3^ Multilabel Plate Reader (Perkin-Elmer) each day in the morning. Obtained growth curves of triplicate cultures were LN(2)-transformed and average generation times were calculated by determination of the derivative of the values between the points of maximal slope (exponential growth phase).

### Microscopic analysis

Images were obtained with a confocal laser-scanning microscope Leica SP5 using a HCX PL APO CS 63.0x1.40 OIL objective, and a Zeiss LSM710 equipped with a C-Apochromat 63x/1.20 W Korr M27 objective. Using the Leica SP5, chlorophyll autofluorescence and YFP fluorescence were excited at 514 nm and detected at 617–687 nm and 520–560 nm, respectively. Using the Zeiss LSM710, chlorophyll autofluorescence and YFP fluorescence were excited at 514 nm and detected at 630–730 nm and 520–550 nm, respectively. Nuclear DNA was stained using the dye Hoechst 33342 (life technologies) at a final concentration of 5 μg / ml and stained cells were visualized by illumination at 405 nm and detection at 410–190 nm. Confocal images were processed further using either the ImageJ 1.45 (Leica images) or LSM Browser 4.2 (Zeiss images) software. 3D projections of the Z-stacks were created using the 3D Project tool (brightest-point projection) in ImageJ 1.45.

## Availability of supporting data

All relevant supporting data can be found within the supplementary files accompanying to this article. Phylogenetic data supporting the results of this article are available in the TreeBASE repository, http://purl.org/phylo/treebase/phylows/study/TB2:S17171.

## Additional files

Additional file 1: Table S1. List of putative CDKA1 and CDKA2 interactor proteins identified by Y2H cDNA library screening. Additional file 2: Figure S1. Confirmation of the candidate CDKA2-interactors identified by a Y2H library screen using pairwise Y2H co-transformation assays. Yeast PJ694-alpha cells were co-transformed with bait (DBD) and prey (AD) plasmid as indicated. Co-transformation was analyzed on medium lacking leucine and tryptophan (-L-T). Co-transformants were tested for their ability to activate the histidine marker gene by assessing yeast growth on medium lacking leucine, tryptophan and histidine (-L-T-H) and for their ability to activate the *LacZ* reporter gene (X-Gal). As a negative control, the *GUS* gene was used. For each combination three independent colonies were screened, of which one is shown. Note that one clone (unknown1) was identified as a general false-positive in the Y2H assay, since it was able to activate itself on medium lacking histidine. Additional file 3: Figure S2. Confirmation of the main nuclear localization of CDKA2-YFP. Confocal laser-scanning image (acquired with a Zeiss LSM710) of a *CDKA2-YFP*-expressing cell during interphase. Green, YFP signal; Red, chlorophyll autofluorescence; Blue, nuclear Hoechst 33342 staining. Scale bars represent 5 μm. Additional file 4: Movie S1. Three-dimensional reconstruction of the Z-stack of confocal images of a *CDKA2-YFP*-expressing cell shown in Figure 5b. The YFP signal is indicated in green. Additional file 5: Movie S2. Three-dimensional reconstruction of the Z-stack of confocal images of a *CDKA2-YFP*-expressing cell shown in Figure 5b. The YFP signal is indicated in green and chlorophyll autofluorescence is indicated in red. Additional file 6: Figure S3. Multiple sequence alignment of the CDK sequences (generated by MUSCLE) used to build the phylogenetic tree shown in Figure 1. Additional file 7: Table S2. Overview of the nCounter code set probe pairs.

## Abbreviations

APC/C: Anaphase promoting complex / cyclosome
CDK: Cyclin-dependent kinase
CLSM: Confocal laser-scanning microscopy
Y2H: Yeast two-hybrid
YFP: Yellow fluorescent protein
