# Supplementary material for: Functional characterization of the diatom cyclin-dependent kinase A2 as a mitotic regulator reveals plant-like properties in a non-green lineage
Source: BMC Plant Biol. 2015 Mar 14;15:86. doi: 10.1186/s12870-015-0469-6 (PMC4392632; doi:10.1186/s12870-015-0469-6)
Supplement: Additional file 1: Table S1. — List of putative CDKA1 and CDKA2 interactor proteins identified by Y2H cDNA library screening. [file 12870_2015_469_MOESM1_ESM.docx]

**Additional file 1: Table S1. List of putative CDKA1 and CDKA2 interactor proteins identified by Y2H cDNA library screening.**

| **BAIT** | **Phatr2 protein ID** | **Description** | **# clones** |
| --- | --- | --- | --- |
|  |  |  |  |
| CDKA1 | 48210 | CYCP1 | 43 |
|  | 49894 | dsCYC7 | 27 |
|  | 21083 | hCdc48 | 3 |
|  | 47279 | bZIP transcription factor | 2 |
|  | 6231 | CYCP6 | 2 |
|  | 45580 | Coronin like protein | 1 |
|  | 34224 | ERO1-like protein precursor / fumarate reductase | 1 |
|  | 45307 | Phox-like | 1 |
|  | 42426 | Prolyl-4-hydrolase alpha subunit | 1 |
|  | 20258 | None | 1 |
|  | 49281 | None | 1 |
|  | 48195 | None | 1 |
|  |  |  |  |
| CDKA2 | 53229 | CKS1 | 1 |
|  | EST | unknown1 | 1 |
|  | 28797 | PTD9, delta 9 desaturase | 1 |
|  | 47845 | Sulfatase | 1 |
