# Supplementary material for: Functional characterization of the diatom cyclin-dependent kinase A2 as a mitotic regulator reveals plant-like properties in a non-green lineage
Source: BMC Plant Biol. 2015 Mar 14;15:86. doi: 10.1186/s12870-015-0469-6 (PMC4392632; doi:10.1186/s12870-015-0469-6)
Supplement: Additional file 2: Figure S1. — Confirmation of the candidate CDKA2-interactors identified by a Y2H library screen using pairwise Y2H co-transformation assays. Yeast PJ694-alpha cells were co-transformed with bait (DBD) and prey (AD) plasmid as indicated. Co-transformation was analyzed on medium lacking leucine and tryptophan (-L-T). Co-transformants were tested for their ability to activate the histidine marker gene by assessing yeast growth on medium lacking leucine, tryptophan and histidine (-L-T-H) and for their ability to activate the LacZ reporter gene (X-Gal). As a negative control, the GUS gene was used. For each combination three independent colonies were screened, of which one is shown. Note that one clone (unknown1) was identified as a general false-positive in the Y2H assay, since it was able to activate itself on medium lacking histidine. [file 12870_2015_469_MOESM2_ESM.docx]

**
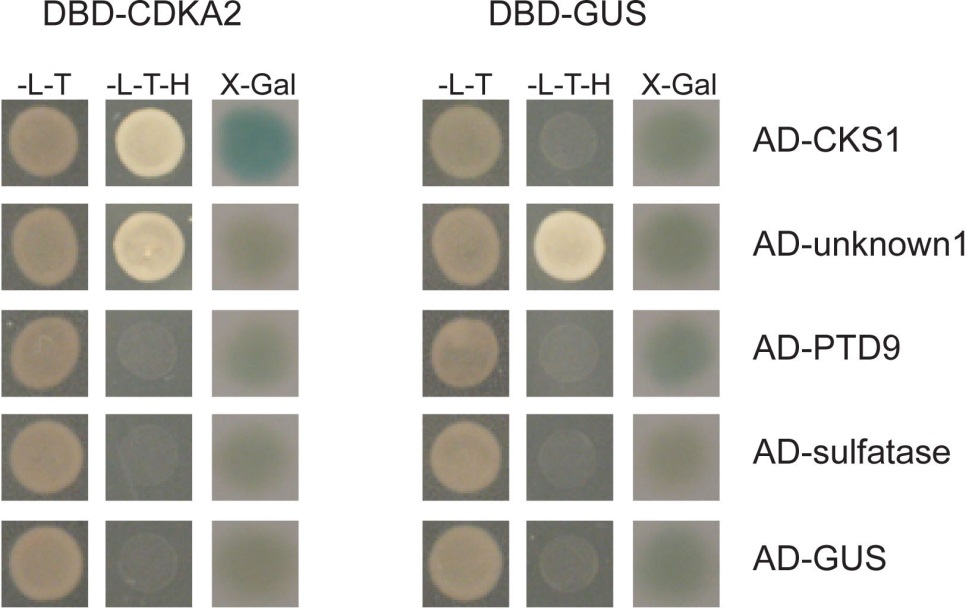
**

**Additional file 2: Figure S1.** **Confirmation of the candidate CDKA2-interactors identified by a Y2H library screen using pairwise Y2H co-transformation assays.** Yeast PJ694-alpha cells were co-transformed with bait (DBD) and prey (AD) plasmid as indicated. Co-transformation was analyzed on medium lacking leucine and tryptophan (-L-T). Co-transformants were tested for their ability to activate the histidine marker gene by assessing yeast growth on medium lacking leucine, tryptophan and histidine (-L-T-H) and for their ability to activate the *LacZ* reporter gene (X-Gal). As a negative control, the *GUS* gene was used. For each combination three independent colonies were screened, of which one is shown. Note that one clone (unknown1) was identified as a general false-positive in the Y2H assay, since it was able to activate itself on medium lacking histidine.
