# Supplementary material for: Functional characterization of the diatom cyclin-dependent kinase A2 as a mitotic regulator reveals plant-like properties in a non-green lineage
Source: BMC Plant Biol. 2015 Mar 14;15:86. doi: 10.1186/s12870-015-0469-6 (PMC4392632; doi:10.1186/s12870-015-0469-6)
Supplement: Additional file 3: Figure S2. — Confirmation of the main nuclear localization of CDKA2-YFP. Confocal laser-scanning image (acquired with a Zeiss LSM710) of a CDKA2-YFP-expressing cell during interphase. Green, YFP signal; Red, chlorophyll autofluorescence; Blue, nuclear Hoechst 33342 staining. Scale bars represent 5 μm. [file 12870_2015_469_MOESM3_ESM.docx]

**
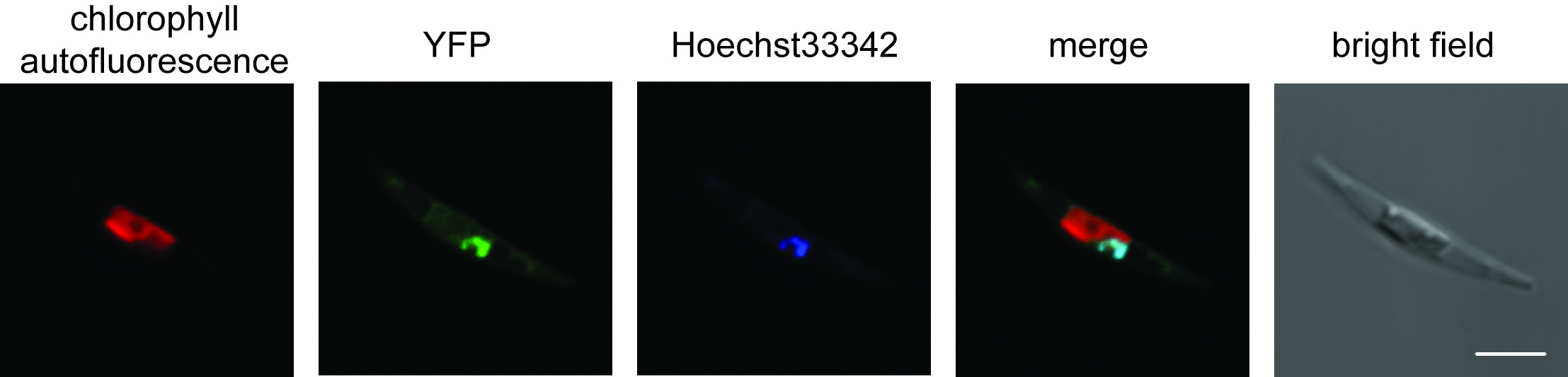
**

**Additional file 3: Figure S2. Confirmation of the main nuclear localization of CDKA2-YFP.**

Confocal laser scanning image (acquired with a Zeiss LSM710) of a *CDKA2-YFP*-expressing cell during interphase. Green, YFP signal; Red, chlorophyll autofluorescence; Blue, nuclear Hoechst33342 staining. Scale bars represent 5µm.
