# Supplementary material for: Functional characterization of the diatom cyclin-dependent kinase A2 as a mitotic regulator reveals plant-like properties in a non-green lineage
Source: BMC Plant Biol. 2015 Mar 14;15:86. doi: 10.1186/s12870-015-0469-6 (PMC4392632; doi:10.1186/s12870-015-0469-6)
Supplement: Additional file 6: Figure S3. — Multiple sequence alignment of the CDK sequences (generated by MUSCLE) used to build the phylogenetic tree shown in Figure 1. [file 12870_2015_469_MOESM6_ESM.pdf]

		20		40		60		80	
Phatr_CDKC2	FTKKHQVGQG	TYGSVFVGQD	KTIVALKRIN	TKEENGFPIT	ALREVKILKV	LHPNIVTLKE	IVVFMVFEYL	EDLTGVLEVK	80
Thaps_CDKC2	FQKKHQVGEG	TYGSVFVGAD	KTVVALKRIN	TEEENGFPIT	AIREVKILKA	LHDNIVQLKE	IVVFMVFEYL	EDLTGIIEIK	80
Outgroup	FEKLNRIGEG	TYGIVYRARD	TTIVALKKVR	MDEKDGIPIS	SLREITLLLR	LHPNIVELKE	VVIFLVMGYC	EDLASLLEVK	80
Phatr_CDKC1	YERQEQIGEG	TYGQVYRARC	KTVVALKKMR	MHGGYGMPLQ	FIREIKILKK	LHPNLLQMIE	VVLFLVLEYV	SDLTGLMDVK	80
Thaps_CDKC1	YCRLEQIGEG	TYGQVYRAKC	LTMVALKKIR	LQSGHGIPPT	VLREIKILKK	LHKAMVKMYE	VVLFLSLEYI	SDLTGLLDVK	80
Ostta_CDKC	YEKLEQIGEG	TYGQVYMARC	KTIVALKKIR	MDEKEGFPIT	AIREIKILKK	LHRNVVDLKE	IVIYLVFEYM	DDLTGLAEIK	80
Arath_CDKC1	FEKLEQIGEG	TYGQVYMAKE	ITIVALKKIR	MDEREGFPIT	AIREIKILKK	LHENVIQLKE	IVIYMVFEYM	DDLTGLADIK	80
Arath_CDKC2	FEKLEQIGEG	TYGQVYMAKE	ITIVALKKIR	MDEREGFPIT	AIREIKILKK	LHENVIHLKE	IVIYMVFEYM	DDLTGLADIK	80
Arath_CDKE1	YNLVGKIGEG	TYGLVFLART	KPPIAIKKFK	QSDGDGVSPT	AIREIMLLRE	IHENVVKLVN	VILYLAFDYA	EDLYEIIRVK	80
Phatr_CDKD1	YKVLSALGQG	TWGVVHKAEQ	KSVVAIKKIK	SEPEEGVNFT	AVREIKLLRE	FHENIVELVD	CFVCLVYECA	FDLEKILGTK	80
Thaps_CDKD1	YEVIKALGQG	TWGVVHMAKQ	RTIVALKKIK	SEPEEGVNFT	AIREIKLLRE	FHENIIELVD	VFVCLVYECA	DDLEKILKTK	80
Arath_CDK7	YAKLSFLGEG	QFATVYKARD	TTIVAVKKIK	KGARDGINRT	ALREIKILQE	LHENIIGLVD	VFVSLVFDFM	DDLEVIIKIK	80
Xenla_CDK7	YEKLDFLGEG	QFATVYKARD	KTIVAIKKIK	LGANDGINRT	ALREIKLLQE	LHPNIIGLLD	AFISLVFDFM	EDLEVIIKIK	80
Homsa_CDK7	YEKLDFLGEG	QFATVYKARD	KTIVAIKKIK	LGAKDGINRT	ALREIKLLQE	LHPNIIGLLD	AFISLVFDFM	EDLEVIIKIK	80
Ostta_CDKD	YERGRTLGEG	TYGVVHEARV	KTVVAIKKIR	LGLKEGVNFT	AIREIKLLQE	IHEHVIELVD	VFLNLVFEYC	GDLEMVIKVK	80
Arath_CDKD1;1	YLKREVLGQG	TYGVVFKATD	TNTVAIKKIR	LGEKEGVNVT	ALREIKLLKE	LHPHIIELID	AFLHIVFEFM	EDLEAVIRVK	80
Arath_CDKD1;2	YLRRQILGEG	TYGVVYKATD	TTTVAVKKIR	LGQKEGVNFT	ALREIKLLKE	LHPHIVELID	AFLHLVFEYM	QDLEAVIRIK	80
Drome_CDK4	YQELNIIGEG	AYGTVYRARD	VTIVALKKVR	ISNENGVPMS	TLREISLLKQ	LHANIVKLYE	VCILLVFEHV	EDLSDLIDIQ	80
Homsa_CDK6	YECVAEIGEG	AYGKVFKARD	LNFVALKRVR	VQGEEGMPLS	TIREVAVLRH	LHPNVVRLFD	VCLTLVFEHV	DDLTTYLDIK	80
Xenla_CDK4	YEPVAEIGVG	AYGTVYKARD	LSFVALKNVR	VQNENGLPLS	TVREVTLLKR	LHPNIVKLMD	VCVTLVFEHV	DDLKTYLSIK	80
Homsa_CDK4	YEPVAEIGVG	AYGTVYKARD	PSFVALKSVR	VPGGGGLPIS	TVREVALLRR	LHPNVVRLMD	VCVTLVFEHV	DDLRTYLDIK	80
Cyame_CDKB	FRRLEVLGQG	TYGTVYKALD	LTIVALKKTT	LSDDEGIPAT	TLREVSILRA	LCENIVKLID	VILYLVFEYA	EDLKQYMNAK	80
Arath_CDKB2;1	FEKLEKVGEG	TYGKVYRARE	KTIVALKKTR	LHDEEGVPST	TLREISILRM	LDPHVVRLMD	VQLYLVFEYM	DDVKKFIRIK	80
Arath_CDKB2;2	FEKLEKVGEG	TYGKVYRARE	KTIVALKKTR	LHDEEGVPPT	TLREISILRM	LDPHIVRLMD	VQLYLVFEYV	DDLKKFIRVK	80
Chlre_CDKB	YEKIEKIGEG	TYGKVYKARD	ITLVALKKCR	LEEEEGVPST	TLREISLLQM	LSNHIVKLLC	VELYLVFEYL	NDMKKWMDIK	80
Arath_CDKB1;1	YEKLEKVGEG	TYGKVYKAME	KTLVALKKTR	LEDEEGIPPT	ALREISLLQM	LTSYVVRLLC	VELYLVFEYL	DDLKKFIDIQ	80
Arath_CDKB1;2	YEKLEKVGEG	TYGKVYKAME	KTLVALKKTR	LEDEEGIPPT	ALREISLLQM	LQSYIVRLLC	VELYLVFEYL	DDLKKFIDVQ	80
Micsp_CDKB	YEKLEKIGQG	TYGKVYKARE	RTLVALKKTR	LEEEEGVPST	ALREVSLLQM	LSAFVVRLLK	VELYLVFEYL	DDLKGFMDVQ	80
Micpu_CDKB	YEKLEKIGQG	TYGKVYKARD	RTLVALKKTR	LEEEEGVPST	ALREVSLLQM	LSAFIVRLLK	VELYLVFEYL	DDLKNFMDVQ	80
Ostlu_CDKB	YEKLEKIGQG	TYGKVYKARE	RNLVALKKTR	LEEEEGVPST	ALREVSLLQM	LSPYIVRLLR	VELYLVFEFL	DDLKQYMDVQ	80
Ostta_CDKB	YEKLEKIGQG	TYGKVYKARD	RTLVALKKTR	LEEEEGVPST	ALREVSLLQM	LSPYIVRLLR	VELYLVFEFL	DDLKQFMDVQ	80
Cyame_CDKA	YQRLERIGEG	TYGVVYRARD	RTLVALKKIR	LEEEEGVPST	ALREIAILRE	LQPNVVRLLD	VVLYLVFEHL	DDLKRYMDAK	80
Physo_CDKA2	YQRIEKIGEG	TYGVVYKSLD	LTVVALKRIR	LEEDDGIPST	ALREISVLRE	LHPNIVSLLD	CLLFLVFEFM	DDLKRFMEIK	80
Ectsi_CDKBlike	YTKQEKAGQG	TYGVVYKSWD	NTFVALKKIK	VEEDDGIPGT	ALREISLLKE	LHPNIVELKN	CVLWLIFEWV	DDLKRYMNIK	80
Nanga_CDKA2	YQKIEKLGEG	TYGVVYKARD	KTIVALKRIR	LEEDEGIPST	ALREISLLQE	LHPCIVELLD	CVLYLVFEFV	DDLKKYMEVK	80
Ectsi_CDKB	YQKIEKLGEG	TYGVVYKAKD	RTIVALKRIR	LEEDEGIPST	ALREISLLRE	LHPNIVELKD	CVLYLIFEFV	DDLKKYMEVK	80
Phatr_CDKA2	YHKIEKLGEG	TYGVVYKALD	RTIVALKRIR	LEEDEGIPST	ALREISLLRE	LHPNIVDLKD	CVLYLVFEFL	DDLKKYMEIK	80
Thaps_CDKA2	YQKIEKLGEG	TYGVVYKARD	RTTVALKRIR	LEEDEGIPST	ALREISLLRE	LHENIVDLKD	CVLYLVFEFL	DDLKKALEVK	80
Fracy_CDKA2	YQKIEKLGEG	TYGVVYKALD	RTIVALKRIR	LEEDEGIPST	ALREISLLRE	LHENVVELMD	CVLYLVFEFL	DDLKKYMEVK	80
Nanga_CDKA1;2	FTNIQQIGEG	TYGIVYKATT	SNLIALKQIR	LAEDEGVPST	AIREISLLKE	LHPNVVTLKD	VILYLVFEYL	DDLKRYMDVK	80
Physo_CDKA1;2	YQKVEKVGEG	TYGIVYKARD	LTIVALKKIR	LEDEEGIPST	AMREISLLKE	LHPNVVYLYD	AVLYLVFEFV	EDLKRCLEVK	80
Sacce_CDC28	YKRLEKVGEG	TYGVVYKALD	LPVVALKKIR	LEEDEGVPST	AIREISLLKE	LDDNIVRLYD	IVLYLVFEFL	DDLKRYMEVK	80
Ectsi_CDKA	YQKLEKIGEG	TYGVVYKAKD	RTIIALKKIR	LEEDEGIPST	AIREISLLKQ	LHPNIVRL--	----------	----------	58
Nanga_CDKA1;1	YQKLEKIGEG	TYGVVFKAKD	RTILALKKIR	LEEDEGIPST	AIREISLLKE	LHPNIVRLYD	VVLTLVFEYL	DDLKKYLDLQ	80
Physo_CDKA1;1	YQKLEKIGEG	TYGVVYKAKD	RTVIALKKIR	LEEDEGIPST	AIREISLLKE	LHCNIVRLYN	IVLTLVFEYL	DDLKKYLDLK	80
Thaps_CDKA1	YQKMEKIGEG	TYGVVYKAKD	RSIIALKKIR	LEEDEGIPST	AIREISLLKE	LHPNIVRLYD	VVLTLVFEFL	DDLKKYLDLK	80
Phatr_CDKA1	YQRMEKIGEG	TYGVVYKAKD	RTIIALKKIR	LEEDEGIPST	AIREISLLKE	LHPNIVRLYD	VVLTLVFEFL	DDLKKYLDLK	80
Fracy_CDKA1	YQKMEKIGEG	TYGIVYKAKD	RTIIALKKIR	LEEDEGIPST	AIREISLLKE	LHPNIVRLYD	VVLTLVFEFL	DDLKKYLDLK	80
Drome_CDK2	FEKIEKIGEG	TYGVVYKGRN	RTIVAMKKIR	LEDDEGVPST	AIREISLLKE	LHENIVCLED	VLIYLIFEFL	SDLKKYMDVR	80
Xenla_CDK1	YTKIEKIGEG	TYGVVYKGRH	KTVVAMKKIR	LEEEEGVPST	AIREISLLKE	LHPNIVCLLD	VLLYLIFEFL	SDLKKYLDVK	80
Homsa_CDK1	YTKIEKIGEG	TYGVVYKGRH	KTVVAMKKIR	LEEEEGVPST	AIREISLLKE	LHPNIVSLQD	VLLYLIFEFL	SDLKKYLDVK	80
Schpo_CDC2	YQKVEKIGEG	TYGVVYKARH	KSIVAMKKIR	LEESEGVPST	AIREISLLKE	VDENCVRLLD	ILLYLVFEFL	DDLKKYMDVQ	80
Xenla_CDK2	FQKVEKIGEG	TYGVVYKARN	RTIVALKKIR	LDETEGVPST	AIREISLLKE	LHPNIVKLLD	VILYLVFEFL	NDLKKFMDVK	80
Homsa_CDK2	FQKVEKIGEG	TYGVVYKARN	KTVVALKKIR	XDETEGVPST	AIREISLLKE	LHPNIVKLLD	VILYLVFEFL	HDLKKFMDIK	80
Ostta_CDKA	YEKVAKIGEG	TYGVVYKAKS	LDMVALKRIR	LDDDEGVPST	AIREISLLKE	LHENVVSLLE	VILYLVFEYL	DDLKKHMDIK	80
Ostlu_CDKA	YEKVLKIGEG	TYGVVYKAKN	LDMVALKRIR	LDDEEGVPST	AIREISLLKE	LHENVVSLLE	VILYLVFEYL	DDLKKHMDVK	80
Micsp_CDKA	FEKVEKIGEG	TYGVVYKARD	RDVVALKRIR	LDEEEGVPST	AIREISLLKE	LHENIVSLMD	VILYLVFEFL	DDLKKHLDIK	80
Micpu_CDKA	FEKVEKIGEG	TYGVVYKARN	RNVVALKRIR	LEEEEGVPST	AIREISLLKE	LHENIVSLMD	VILYLVFEHL	DDLKKHLDIK	80
Chlre_CDKA	YEKLERAGEG	TYGVVFKARD	RTIVALKKIR	LEEDEGVPST	AIREISFLKE	LHDNVVRLYD	VLLYLVFEFL	DDLKKQMDIK	80
Arath_CDKA1	YEKVEKIGEG	TYGVVYKARD	KTTIALKKIR	LEEDEGVPST	AIREISLLKE	MHSNIVKLQD	VVLYLVFEYL	DDLKKHMDIK	80

		100		120		140		160	
Phatr_CDKC2	SWSQQLLGVH	YMHVIHRDLK	ASNLLILKIA	DWGLARSWNS	EMLTNKVITL	WYRPPELLLG	YTDKIDMWSV	GCIIAEMFRS	160
Thaps_CDKC2	SWSKQLLGVH	YMHIIHRDLK	SSNLLILKIA	DWGLARSWNS	EMLTNRVITL	WYRPPELLLG	YSTKIDMWSV	GCIIAEMFRG	160
Outgroup	CIVLQVLGLQ	YLHIIHRDLK	VSNLLMVKTA	DFGLARAYGV	PVMTPKVVTL	WYRAPELLLG	QTTSIDMWAV	GCILAELLHR	160
Phatr_CDKC1	CIFRQLLACA	YMHYVHRDIK	SSNILLVKLA	DFGLARNIEP	PFFTNKVITL	WYRPPEILTG	YGPAVDVWSA	GCILAELLGK	160
Thaps_CDKC1	SVVQQLLVLE	FMHYVHRDLK	SSNILIVKLA	DFGLARCLEG	--FTNKVITL	WYRPPELLLG	YGTAVDIWSA	GCILAEVIGR	158
Ostta_CDKC	CYMKQLLGLH	YCHILHRDIK	GSNLLILKLA	DFGLAKPITN	ENLTNRVITL	WYRPPELLLG	YGPSVDMWSA	GCIFAELIGK	160
Arath_CDKC1	CYMKQLLGLH	YCHVLHRDIK	GSNLLILKLA	DFGLARSYSH	DHLTNRVITL	WYRPPELLLG	YGPAIDMWSV	GCIFAELLAK	160
Arath_CDKC2	CYMKQLLGLH	YCHVLHRDIK	GSNLLILKLA	DFGLARSYSH	DHLTNRVITL	WYRPPELLLG	YGPAIDMWSV	GCIFAELLGK	160
Arath_CDKE1	SLLWQLLGLN	YLHIIHRDLK	PSNILVVKIA	DFGLARIYQA	PLLSDNVVTI	WYRAPELLLG	YTSAVDMWAV	GCIFAELLLK	160
Phatr_CDKD1	QHLLSLLAIS	ACHILHRDLK	PDNMLFMKLA	DFGLARMHGT	PKLSPQAITL	WYKPPELLLG	YSSSADMWSV	GCIFAELLRR	160
Thaps_CDKD1	QHLLTLLAIS	ACHILHRDLK	PDNCLFMKLA	DFGLARMYGT	PKLSPQAITL	WYKPPELLLG	YSSAADIWSV	GCIFAELLRR	160
Arath_CDK7	AYAIMTLGLE	YLHILHRDLK	PNNLLVLKIG	DFGLAKSFGS	PNYTHHVVTR	WYRSPELLFG	YGTGVDMWAV	GCILAELMRV	160
Xenla_CDK7	SYMLMTLGLE	YLHILHRDLK	PNNLLLLKLA	DFGLAKSFGS	PNYTHQVVTR	WYRSPELLFG	YGVGVDMWAV	GCILAELLRV	160
Homsa_CDK7	AYMLMTLGLE	YLHILHRDLK	PNNLLLLKLA	DFGLAKSFGS	PNYTHQVVTR	WYRAPELLFG	YGVGVDMWAV	GCILAELLRV	160
Ostta_CDKD	SYARMTLAVA	HCHVLHRDLK	PNNLLILKLA	DFGLARIFGS	PDFTHQVFAR	WYRAPELLLG	YGPGVDVWAV	GCILAELMRK	160
Arath_CDKD1;1	SYLQMILGLE	YCHVLHRDMK	PNNLLILKLA	DFGLARIFGS	PGFTHQVFAR	WYRAPELLFG	YDGAVDVWAA	GCIFAELLRR	160
Arath_CDKD1;2	SYMLMTLGLA	YCHVLHRDMK	PNNLLILKLA	DFGLARLFGS	PNFTHQVFAT	WYRAPELLFG	YGAGVDVWAA	GCIFAELLRR	160
Drome_CDK4	RLSRELLGVD	FLHIIHRDLK	PQNLLVLKIA	DFGLAKTYGS	EMLTSVVVTL	WYRAPEVLL-	YNSTVDIWSA	ACIIFEMFRR	159
Homsa_CDK6	DMMFQLLGLD	FLHVVHRDLK	PQNILVIKLA	DFGLARIYSF	QMLTSVVVTL	WYRAPEVLLQ	YATPVDLWSV	GCIFAEMFRK	160
Xenla_CDK4	DLMKQFLGLE	FLHIVHRDLK	PENILVVKLA	DFGLARIYSC	QMLTPVVVTL	WYRAPEVLLQ	YATPVDVWSA	GCIFAEMFRK	160
Homsa_CDK4	DLMRQFLGLD	FLHIVHRDLK	PENILVVKLA	DFGLARIYSY	QMLTPVVVTL	WYRAPEVLLQ	YATPVDMWSV	GCIFAEMFRK	160
Cyame_CDKB	CFAYQMLGLQ	FCHIMHRDLK	PQNILVIKLA	DFGLGRAFCI	PVYTHEVVTL	WYRAPEILLG	YSTPVDIWSV	GCILAEMIGR	160
Arath_CDKB2;1	SLMYQLCGMA	FCHILHRDLK	PHNLLMLKIA	DLGLARAFTL	PMYTHEILTL	WYRAPEVLLG	YSTAVDMWSV	GCIFAELVNQ	160
Arath_CDKB2;2	CLMYQLCGMA	FCHVLHRDLK	PHNLLMLKIA	DLGLARAFTL	PMYTHEILTL	WYRAPEVLLG	YSTGVDMWSV	GCIFAELVKQ	160
Chlre_CDKB	SMVYQLIGVA	YCHVLHRDLK	PQNLLVLKVA	DLGLGRHFSV	PLYTHEIVTL	WYRAPEVLLG	YATPVDMWSV	GCIFAELVKA	160
Arath_CDKB1;1	KLMFQLCGVA	HCHVLHRDLK	PQNLLLLKIA	DLGLGRAFTV	PLYTHEIVTL	WYRAPEVLLG	YSTGVDMWSV	GCIFAEMVRQ	160
Arath_CDKB1;2	RFMFQLFGVA	HCHVLHRDLK	PQNLLLLKIA	DLGLSRAFTV	PLYTHEIVTL	WYRAPEVLLG	YSTAVDIWSV	GCIFAEMIRQ	160
Micsp_CDKB	DFMYQLCGCA	HIHVMHRDLK	PQNLLVIKVA	DLGLGRAFSV	PVYTHEIVTL	WYRAPEVLLG	YSTPVDIWSV	GCIFAELAKQ	160
Micpu_CDKB	DFMYQLCGCA	HLHVMHRDLK	PQNLLVIKVA	DLGLGRAFSV	PVYTHEIVTL	WYRAPEVLLG	YSTPVDIWSV	GCIFAELAKQ	160
Ostlu_CDKB	NYMYQLCGCA	HLHVMHRDLK	PQNMLVLKIA	DLGLGRAFSV	PVYTHEIVTL	WYRAPEVLLG	YSTPVDMWSV	GCMFAEMAKQ	160
Ostta_CDKB	NYMYQLCGCA	HLHVMHRDLK	PQNMLVLKIA	DLGLGRAFSV	PVYTHEIVTL	WYRAPEVLLG	YSTPVDMWSV	GCMFAEMAKQ	160
Cyame_CDKA	SFLYQLIGVA	YLHILHRDLK	PQNLLILKLA	DFGLARAFGI	PVMTSEVITL	WYRAPEILLG	YAAPVDMWSV	GCIFAEMMRK	160
Physo_CDKA2	SLLYQLLGLA	FSHIMHRDLK	PQNLLVLKIA	DFGLARAFSL	PIYTHEVVTL	WYRAPEILLG	YCPPVDIWSV	GVIFAEMVKK	160
Ectsi_CDKBlike	SYMFQMMALE	FCHIMHRDLK	PQNLLVLKIA	DFGLARAFCP	PVLTHEVVTI	WYRAPEILLG	YAPPLDLWSV	GTILAEMVKT	160
Nanga_CDKA2	SFLFQIFGLA	FCHIMHRDLK	PQNLLVLKIA	DFGLARAFVP	PILTHEVVTL	WYRPPEILLG	YAPPVDVWAC	GAIFVELLKR	160
Ectsi_CDKB	SYLFQMVGLE	FCHVMHRDLK	PQNLLVLKLA	DFGLARAFCP	PILTHEVVTL	WYRAPEILLG	YAPPVDLWAV	GTILVEMVKR	160
Phatr_CDKA2	SYTFQCLGLA	FCHVMHRDLK	PQNLLVLKIA	DFGLARAFCP	PILTHEVVTL	WYRPPEILLG	YAPPMDVWAI	GAILVEMIKR	160
Thaps_CDKA2	SYLFQMCGLA	FCHVMHRDLK	PQNLLVLKLA	DFGLARAFCP	PILTHEVVTL	WYRPPEILLG	YAPPVDVWAI	GTIFVEMVKR	160
Fracy_CDKA2	SYLFQICGLA	FCHVMHRDLK	PQNLLVLKLA	DFGLARAFCP	PILTHEVVTL	WYRPPEILLG	YAPPVDVWAI	GAIFVEMVKR	160
Nanga_CDKA1;2	SYLHQMIGVA	FCHVLHRDLK	PQNLLILKLA	DFGLARAFNV	PLYTREVVTL	WYRAPEILLG	YSTPVDTWSI	GCIFAEMLKE	160
Physo_CDKA1;2	SYLYQLLGIA	FCHVLHRDLK	PQNLLILKLG	DFGLAREYGV	PLYTHEVVTL	WYRAPEVLLG	YSTPVDSWSI	GCIFAEMVKQ	160
Sacce_CDC28	KFMMQLCGIA	YCHILHRDLK	PQNLLILKLG	DFGLARAFGV	PLYTHEIVTL	WYRAPEVLLG	YSTGVDTWSI	GCIFAEMCRK	160
Ectsi_CDKA	----------	-----HRDLK	PQNLLILKLA	DFGLARAFGI	PVYTHEVVTL	WYRAPDVLMG	YSTPVDIWSI	GCIFAEMAGR	123
Nanga_CDKA1;1	SFLYQLLGVA	FCHVLHRDLK	PQNLLILKLA	DFGLARAFGI	PVYTHEVVTL	WYRAPDVLMG	YSTPVDIWSI	GCIFAEMAGR	160
Physo_CDKA1;1	SFLYQLLGIA	YCHVLHRDLK	PQNLLILKLG	DFGLARAFGI	PVYTHEVVTL	WYRAPDVLMG	YSTPVDIWSV	GCIFAEMAGG	160
Thaps_CDKA1	SFLYQLLGVA	YCHVLHRDLK	PPNLLILKLA	DFGLARAFGI	PVYTHEVVTL	WYRAPDVLMG	YSTPVDIWSV	GCIFAEMVGR	160
Phatr_CDKA1	SFLYQLLGVA	YCHVLHRDLK	PPNLLILKLA	DFGLARAFGI	PVYTHEVVTL	WYRSPDVLMG	YSTPVDIWSV	GCIFAEMAGR	160
Fracy_CDKA1	SFLYQLLGVA	YCHVLHRDLK	PPNLLILKLA	DFGLARAFGI	PVYTHEVVTL	WYRAPDVLLG	YSTPVDIWSV	GCIFAEMAGR	160
Drome_CDK2	SYLYQITAIL	FCHVLHRDLK	PQNLLIIKVA	DFGLGRSFGI	PVYTHEIVTL	WYRAPEVLLG	YSCPVDIWSI	GCIFAEMARK	160
Xenla_CDK1	SYLYQILGIV	FCHVLHRDLK	PQNLLIIKLA	DFGLARAFGI	PVYTHEVVTL	WYRAPEVLLG	YSTPVDVWSI	GTIFAEIAKK	160
Homsa_CDK1	SYLYQILGIV	FCHVLHRDLK	PQNLLIIKLA	DFGLARAFGI	PIYTHEVVTL	WYRSPEVLLG	YSTPVDIWSI	GTIFAELAKK	160
Schpo_CDC2	KFTYQLVGVN	FCHIIHRDLK	PQNLLILKLA	DFGLARSFGV	PLYTHEIVTL	WYRAPEVLLG	YSTGVDIWSV	GCIFAEMIRS	160
Xenla_CDK2	SYLFQLLGLA	FCHVLHRDLK	PQNLLIIKLA	DFGLARAFGV	PVFTHEVVTL	WYRAPEILLG	YSTAVDIWSL	GCIFAEMIRR	160
Homsa_CDK2	SYLFQLLGLA	FCHVLHRDLK	PQNLLIIKLA	DFGLARAFGV	PVYTHEVVTL	WYRAPEILLG	YSTAVDIWSL	GCIFAEMVRR	160
Ostta_CDKA	GYVYQICGIA	FCHVLHRDLK	PQNLLILKLA	DFGLARAFGI	PVYTHEVVTL	WYRAPEILLG	YSTPVDVWSI	GCIMAEMVHA	160
Ostlu_CDKA	GYVYQICGIA	FCHVLHRDLK	PQNLLILKLA	DFGLARAFGI	PVYTHEVVTL	WYRAPEILLG	YSTPVDVWSI	GCIFAEMIGK	160
Micsp_CDKA	GYLYQMCGIA	YCHVLHRDLK	PQNLLVLKLA	DFGLARAFGI	PLYTHEVVTL	WYRSPEILLG	YSTPVDVWSI	GCIFAEMIHA	160
Micpu_CDKA	GYLYQMCGVA	YCHVLHRDLK	PQNLLVLKLA	DFGLARAFGI	PVYTHEVVTL	WYRSPEILLG	YSTPVDVWSI	GCIFAEMIHA	160
Chlre_CDKA	MYMWQMLGIA	FCHILHRDLK	PQNLLILKLA	DFGLARAFGI	PVYTHEVVTL	WYRAPEILLG	YSTPVDIWSI	GCIFAEMVHR	160
Arath_CDKA1	TYLYQILGIA	YCHVLHRDLK	PQNLLILKLA	DFGLARAFGI	PVFTHEVVTL	WYRAPEILLG	YSTPVDIWSV	GCIFAEMIQK	160

```
                         180                                200                                220
                          |                                  |                                  |
Phatr_CDKC2    GFLKGSNEAT QLLIFRTCGH PTKEWPSLGD KRLWFPLKLP PAIELIKLMA LNPRWSAEHA LDAEYF 226
Thaps_CDKC2    GLLKGSSEAS QLLIFRVMGH PTTEWPNINK MPLWLPLRLP AAMDMINLLA YNPRWSAAQA LTAEWF 226
Outgroup       PLLPGTSEIH QILIVQLLGT PSENWPGFSK LPLVYSLKFP LGLRLLFLFM YDPRATAGDC LESSYF 226
Phatr_CDKC1    PLLPGKSDLE QLLIADMLGT PAPDWDYLSS MKRVLDLYAA IAMNLLKLLA WDPRLTATGA LQNKYF 226
Thaps_CDKC1    PMFTGKTDMD QLLIFDLIGT PTDKWEGFRQ LKLIIDLKYS KALNLLKLLE LDPRISARGA LTHRYF 224
Ostta_CDKC     PILPGKSEME QMIIFKLCGS PTPEWPDADK LPYAFTLVFS FAKDLLKFLT LDPRISAKDA LMHDWF 226
Arath_CDKC1    PILPGKNEQE QLKIFELCGS PDEKWPGVSK MPWFFKVFFR FALELLKMLV LDPRISAKDA LDAEYF 226
Arath_CDKC2    PILPGKTENE QLKIYELCGS PDESWPGVSK MPWYMKVIYR FALELLKMLV LDPRICAKDA LDAEYF 226
Arath_CDKE1    PLFQGEAQLD QLKIFKILGH PTMDWPTLVN LPHWIQLVHL QAYDLLKMLE YDPRITASQA LEHEYF 226
Phatr_CDKD1    PFLQGQTDLS QLTIFTVFGT PNETWADFQT LPLCWDFIFT AAISLLSILV LDPRFTATQC LSHPYF 226
Thaps_CDKD1    PFLQGQSDVS QLTIFQVFGT PTETWPDHNT LPLCWDFIFT ACLSLLSMLV LDPRITANAA LLHPYF 226
Arath_CDK7     PFMPGDSDLD QLRIFSTLGT PTEAWPHLSK LHDYFRLIFT ALIHLMRLFA MNPRVSCREA LSMPYF 226
Xenla_CDK7     PFLPGDSDLD QLRIFETLGT PTEEWPGMSS LPDYFKLIFI ALLELLGLFT FNPRCTASQA LRKRYF 226
Homsa_CDK7     PFLPGDSDLD QLRIFETLGT PTEEWPDMCS LPDYFKLIFS ALLDLIGLFL FNPRITATQA LKMKYF 226
Ostta_CDKD     PFFAGSSDID QLKVYAALGT PTETWPGVSA LPDFFILTFP ESLDLLRMLE YDPRITAAQA LEHPYF 226
Arath_CDKD1;1  PFLQGNSDID QLKIFAAFGT PKADWPDMIC LPDYYQLLLP VALDLLKMFT YDPRISIQQA LKHRYF 226
Arath_CDKD1;2  PFLPGSTEID QLKIFQAFGT PVPSWSDMIY LPDYFSLIFP AALDLLKMFI YDPRITIQQA LDHRYF 226
Drome_CDK4     ALFPGTSEKN QLRIFELTGR PTEQWPQTIS VALEFPPFCP LADDLLKMLS YDLRPSALAC LEHDYF 225
Homsa_CDK6     PLFRGSSDVD QLKILDVIGL PGEEWPRDVA LPRQFHIFVT IGKDLLKCLT FNPRISAYSA LSHPYF 226
Xenla_CDK4     PLFCGNSEAD QLKIFDIIGL PSEEWPVDVT LPRSFSVFVP IGADLLAMLT FSPRISASDA LLHPFF 226
Homsa_CDK4     PLFCGNSEAD QLKIFDLIGL PPEDWPRDVS LPRGFPVVVP MGAQLLEMLT FNPRISAFRA LQHSYL 226
Cyame_CDKB     SLFCGESEIE QLAIFRVLGT PNEQWPSVVE LRDWFPLILP LGCKLLEMLQ LDPRITAADA LRHPFF 226
Arath_CDKB2;1  AIFQGDSELQ QLHIFKLFGT PNEEWPGVST LKNWYPLAVP LGVDLLKMLQ YEPRISAKMA MEHPYF 226
Arath_CDKB2;2  AIFAGDSELQ QLRIFRLLGT PNEEWPGVSK LKDWYPLAVP LGLDLLKMLE YEPRISAKKA MEHPYF 226
Chlre_CDKB     PLFPGDSEYQ QLHIFKLLGT PSEDWPGVTK LRDWWPLIFP LGIDLMRCFA YDPRISAKEA INHPYF 226
Arath_CDKB1;1  ALFPGDSEFQ QLHIFRLLGT PTEQWPGVST LRDWYPLAVP LGVDLLKMLK YNPRISAKTA LDHPYF 226
Arath_CDKB1;2  ALFPGDSEFQ QLHIFRLLGT PTEQWPGVMA LRDWYPLAVP LGIDLLQMLK YNPRISAKAA LDHPYF 226
Micsp_CDKB     PLFPGDSELQ QLHIFKLLGT PSEDWPGVTR LRDWFPLVIP LGIDLLKMLV YDPRIHATEA LEHPYF 226
Micpu_CDKB     PLFPGDSELQ QLHVFKLLGT PSEEWPGVTR LRDWFPLVIP LGIDLMKMLV YDPRIHATEA LEHPYF 226
Ostlu_CDKB     PLFPGDSELQ QLHIFKLLGT PSEQWPGVSN LRDWFPLVIP LGIDLLKLLV YDPRIHATDA LEHPYF 226
Ostta_CDKB     PLFPGDSELQ QLHIFKLLGT PSEQWPGVSN LRDWFPLLIP LGIDLLKLLV FDPRIHATDA LEHPYF 226
Cyame_CDKA     ALFPGDSEID QLKIFRALGT PSEEWPGVSQ LPDYFPIVLA GALDLLRLLV YDPRITARQA LMHPYF 226
Physo_CDKA2    PLFTGDSEID QLRIFRTLGT PNESWPGVTK LRDYFPLLFP LGLHLLSMLR YDPRISAKEA LRHPYF 226
Ectsi_CDKBlike PMFPGDSEID EIKIFRVLGT PTESWPNVTD LDDWFPLEYA LGINMLQLLA YDPRLSAKRA LKHPFF 226
Nanga_CDKA2    AMFQGDSEVD QLKIFRSLGT PSEEWPGVTA LQDWFPLYCP IGLDLLKLVV LDPRISAKTA LYHRYF 226
Ectsi_CDKB     PLFPGDSEID EIKIFQLLGT PNEEWPNVTD LQDWFPLRSQ MGLDLLKLLY YDPRISAKRA LEHAFF 226
Phatr_CDKA2    PLFPGDSEID QLKIFRQLGT PREDWPGVTQ LQDWFPFVLD LGLELLTILA YDPRITAKES LDHAYF 226
Thaps_CDKA2    PLFPGDSEID ELKIFRQLGT PNEEWPGVTA LQDWFPFFLD TAVDLLRLLA YSPRITAKDT LNHPYF 226
Fracy_CDKA2    PLFPGDSEID QLKIFRLLGT PNEEWPGVTH LPDWFPYVTE LGMDLLRFLF YDPRITAKDS LNHPYF 226
Nanga_CDKA1;2  PLFPGDSEID ELRIFRVLGT PNDEWPKVTE LPNYFPLNVS LGLDLLRLLT YEPRITCREA QDHAYF 226
Physo_CDKA1;2  PLFPGDSEID ELRIFRVLGT PNEAWPGVST LPDYFPLVVP LGLDLLRLLV YDPRISARAA MSHPWF 226
Sacce_CDC28    PIFSGDSEID QIKIFRVLGT PNEAWPDIVY LPDFFPLVVP LGIDLLKLLA YDPRISARRA AIHPYF 226
Ectsi_CDKA     PLLTGTSESD QLRIFRQMGT PTPIYPGLQE LPDY--VGGP AYTKLMRRIR WGEDSNSDEE VSKPAL 187
Nanga_CDKA1;1  PLFAGSSESD QLRIFRALGT PTEGYPGIVE LPEFFPWLVP LGIDLLKMLT FDPRVSARDA LSHPFF 226
Physo_CDKA1;1  PLFAGTSEAD QLRIFRLLGT PTMEYPAIID LPEYFPLLVP LGVDLLQMLQ YDPRITAADA MAHPYF 226
Thaps_CDKA1    PLIAGTSEGD QLRIFRLLGT PSPAFPGIVD LPEYLPFLVP LGVDLLKMLQ YDPRITANDA LKHPFF 226
Phatr_CDKA1    PLVAGTSEAD QLRIFRLLGT PKLEYPTINE LPEYMPLLVP LGIDLLRMLQ YDPRITAQAA LEHEYF 226
Fracy_CDKA1    PLVAGTSEDD QLRIFRLLGT PKPSFPGIVD LPDWYPLLVP LGIELLLMLQ YDPRITAQDA LQHRFF 226
Drome_CDK2     PLFQGDSEID QLRMFRILKT PTEDWPGVTS LPDYFPLQLK LGIDLIKMLI YDPRISAKDI LEHPYF 226
Xenla_CDK1     PLFHGDSEID QLRIFRALGT PNNEWPEVES LQDYFPLNVK IGLDLLKMLI YDPRISARKA LLHPYF 226
Homsa_CDK1     PLFHGDSEID QLRIFRALGT PNNEWPEVES LQDYFPLHVK LGLDLLKMLI YDPRISGKMA LNHPYF 226
Schpo_CDC2     PLFPGDSEID EIKIFQVLGT PNEEWPGVTL LQDYFPLVVP GAIELLAMLV YDPRISAKRA LQQNYL 226
Xenla_CDK2     ALFPGDSEID QLRIFRTLGT PDEVWPGVTT MPDYFPFVVP LGRDLLQMLQ YDSRISAKVA LTHPFF 226
Homsa_CDK2     ALFPGDSEID QLRIFRTLGT PDEVWPGVTS MPDYFPFVVP LGRSLLQMLH YDPRISAKAA LAHPFF 226
Ostta_CDKA     PLFPGDSEID ELKIFKTLGT PGETWPEAKQ LPDYFPWLCP LGVDLLSLLQ YSPRISAKYA TQHRWF 226
Ostlu_CDKA     PLFPGDSEID ELKIFKILGT PNETWPEAQE LPDYFPWLCP LGVDLLQMLQ YTPRISAKHA MQHKWF 226
Micsp_CDKA     PLFPGDSEID ELRIFRVLGT PDDGWQGVEQ LPDYFPWICP LGLDLLQMLQ YAPRISARDA CNHRFF 226
Micpu_CDKA     PLFPGDSEID QLRIFRVLGT PDDDWPAVSS LPDYFPWVCP LGIDLLSLLH YAPRVSAREA CEHRFF 226
Chlre_CDKA     PLFPGDSEID QLKIFQLLGT PDEAWAGCSA LPDFFPLAVP LGVDLLRMLV YTPRITASAA MEHAYF 226
Arath_CDKA1    PLFPGDSEID QLKIFRIMGT PYEDWRGVTS LPDYFPLFVP LGVDLLKMLL MDPRINARAA LEHEYF 226
```
