# Supplementary material for: Functional characterization of the diatom cyclin-dependent kinase A2 as a mitotic regulator reveals plant-like properties in a non-green lineage
Source: BMC Plant Biol. 2015 Mar 14;15:86. doi: 10.1186/s12870-015-0469-6 (PMC4392632; doi:10.1186/s12870-015-0469-6)
Supplement: Additional file 7: Table S2. — Overview of the nCounter code set probe pairs. [file 12870_2015_469_MOESM7_ESM.docx]

**Additional file 7: Table S2. Overview of the nCounter code set probe pairs.**

| **Gene** | **Accession** | **Targeted Region** | **Target Sequence** |
| --- | --- | --- | --- |
| CDKA1 | XM_002180023.1 | 90-190 | ACGGTCGTGATTTCTTGTGGTGATCGGTCTCGAGCCGCAGTTCGCCACTCTGCCCGGAACAGACCGGAGATTCTCCTTGGACTTACTTTTTTCACCGTAG |
| CDKA2 | XM_002185292.1 | 220-320 | TTGCCCTCAAGCGTATCCGATTGGAAGTCGAAGACGAAGGCATTCCCTCGACGGCTCTCCGTGAAATATCTCTGTTGCGGGAACTTTCGCACCCGAACAT |
| CYCB1 | XM_002180361.1 | 805-905 | TTAGAGAAAAGTCAATCGCAAATCCTGAGCTCCGAGCGGTGAATAAAAAGTACAGCGGTCATCGATACGGCGGAGTTGCTTCGACCGTTCTGGTATTTGA |
| H4 | XM_002179469.1 | 100-200 | CTATCCGCCGTCTGGCCCGTCGTGGTGGTGTGAAACGTATCTCTGGTCTGATTTACGAAGAGACCCGTGGAGTCCTCAAGGTCTTCCTCGAGAACGTCAT |
| UBI-4 | XM_002182214.1 | 216-316 | TTCAGAAGGAGTCAACTTTACATTTGGTGTTGCGTCTACGTGGAGGAGTCTATGATCCGTCGCTCGCTTTACTGGCCAAGGGATTTAACTGTGACAAGGC |
| RPS | XM_002178225.1 | 396-496 | CTCCGCGCATTTTTGCCCGGTTCCCATTTGACCGGACAGTTGCCCGACGAAGATCTCATTGGAAACAACTTGCAGCTTAAATTCCTCGAAGTCAACCAGG |
| EF1a | XM_002186267.1 | 1498-1598 | CGGGAGTGGACCGCGACGATCCGTCCGGAAAACAATACTAGGTGCTATCACAGGGGCGCGTTTTGGAGAGACGTTCTGCGGAAACACGAATTTAGAATAC |
